# Assessment of changes in gait parameters and vertical ground reaction forces after total hip arthroplasty

**DOI:** 10.4103/0019-5413.32050

**Published:** 2007

**Authors:** P Bhargava, P Shrivastava, SP Nagariya

**Affiliations:** Prof & Head, Dept of Orthopaedics M. Y. Hospital Indore, Dept. of Orthopedics, M.Y Hospital, Indore, India; * Artificial Limb Fitting Center, Dept. of Orthopedics, M.Y. Hospital, Indore, India

**Keywords:** Gait parameters, total hip replacement, vertical ground reaction forces

## Abstract

The principal objectives of arthroplasty are relief of pain and enhancement of range of motion. Currently, postoperative pain and functional capacity are assessed largely on the basis of subjective evaluation scores. Because of the lack of control inherent in this method it is often difficult to interpret data presented by different observers in the critical evaluation of surgical method, new components and modes of rehabilitation. Gait analysis is a rapid, simple and reliable method to assess functional outcome. This study was undertaken in an effort to evaluate the gait characteristics of patients who underwent arthroplasty, using an Ultraflex gait analyzer.

**Materials and Methods:**

The study was based on the assessment of gait and weight-bearing pattern of both hips in patients who underwent total hip replacement and its comparison with an age and sex-matched control group. Twenty subjects of total arthroplasty group having unilateral involvement, operated by posterior approach at our institution with a minimum six-month postoperative period were selected. Control group was age and sex-matched, randomly selected from the general population. Gait analysis was done using Ultraflex gait analyzer. Gait parameters and vertical ground reaction forces assessment was done by measuring the gait cycle properties, step time parameters and VGRF variables. Data of affected limb was compared with unaffected limb as well as control group to assess the weight-bearing pattern. Statistical analysis was done by‘t’ test.

**Results:**

Frequency is reduced and gait cycle duration increased in total arthroplasty group as compared with control. Step time parameters including Step time, Stance time and Single support time are significantly reduced (*P* value <.05) while Double support time and Single swing time are significantly increased (*P* value <.05) in the THR group. Forces over each sensor are increased more on the unaffected limb of the THR group as compared to the control group. Vertical ground reaction force variables are also altered.

**Conclusion:**

Significant changes (*P* value <.05) in gait parameters and vertical ground reaction forces show that gait pattern is not normalized after THR and weight-bearing is not equally shared by both hips. Patient walks with residual antalgic gait even after surgery, which results in abnormal loading around hip joints and the integrity of the prosthesis fixation could be compromised.

The principal objectives of arthroplasty are relief of pain and enhancement of range of motion. Previous studies on arthroplasty have focused either on surgical technique, postoperative management or radiographic assessment but not on its functional outcome. Most of the patients postoperatively are satisfactorily relieved from pain as well as have acceptable improvement in functions and mobility. But evaluation of these subjective variables is questionable and accurate assessment is difficult. Joint motion is a functional variable of the hip, which returns to normal after months postoperatively. Continued atypical joint motion leads to abnormal loading and further osteoarthritis. Thus another objective method, which is a reliable indicator for functional assessment of a patient's gait, would be of great value.

Gait analysis is simply a measurement system that allows the user to monitor and analyze human locomotion. Quantification of human motion by the measurement of joint motion, electromyography activity of muscle and the forces both created by and acting upon the body can be precisely recorded and evaluated. These measurements may be coordinated in time to allow comparison between modes of evaluation, creating an accurate assessment of patients' ambulatory ability.

Measures determined from gait parameters and vertical ground reaction forces have been used to quantify abnormal limb loading for individuals after hip arthroplasty.^1^–^3^ Gait parameters and vertical ground reaction forces provide indirect information about internal joint loading because peak ground reaction forces coincide with the timing of peak loads on the femur and hip joint during gait.^4^,^5^

The purpose of the study was to compare the selected measures from vertical ground reaction force variables and gait parameters of hip replacement patients to a normal healthy age and sex-matched control group. It was hypothesized that there would be no significant differences between normal individuals and hip replacement subjects for gait parameters and vertical ground reaction forces and weight-bearing would be equally shared by the both hips. We hope that the results of this study will further encourage the use of objective testing in clinical settings.

## MATERIALS AND METHODS

Twenty pain-free individuals (mean age-51.6 years), operated by posterior approach for total hip arthroplasty (THA) were included in the study. The control group subjects were randomly selected from the general population and were age and sex-matched to the subjects in the Total Arthroplasty group so that the control group would exhibit a comparable gait pattern to that age group. All the subjects were at least six-month post surgery (range 6-51 months) and had completed their prescribed rehabilitation regimen.

Subjects with a medical condition that would compromise their ability to walk were excluded from the study. Only individuals with unilateral degenerative hip disease participated. Secondary involvement of the lower limb joint was ruled out based on a clinical assessment using the guidelines for Osteoarthritis classification of the American College of Rheumatology.^6^,^7^

### Procedures

Ultraflex (Gait analysis system) by Infrotronics Medical Industrial Engineering was used for data collection. It has CDG Computer Dynography. The complete ultraflex gait analysis system consists of the following parts:

#### CDG Shoes with sensors:

**Figure 1:**
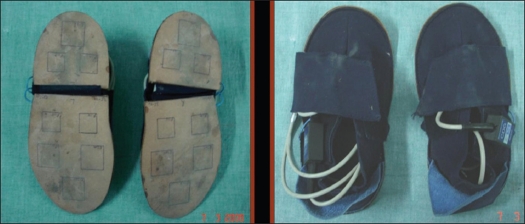
CDG shoes with sensors

CDG shoes are designed to measure and record the normal forces under the foot while walking. Each shoe contains 8-load sensor at the sole. Cable attached to shoes transfers the normal forces data to the ultraflex unit for recording [Figure 1].

**Figure:**
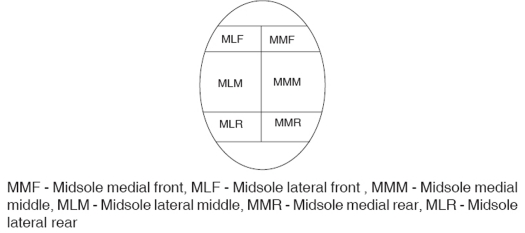


#### Measurements unit:

The ultraflex unit is a portable measurement unit that records normal ground reaction forces while walking. All measurement data will be stored in to the memory card while conducting a new measurement.

#### Ultraflex Optical link cable:

It is glass fiber cable. Its main function is for high-speed data transfer.

#### Cords:

Used to connect ultraflex measurement unit to the computer used for data analysis.

#### Straps:

Used to fix the cord to the body so that patients have no problem in walking.

### Method of data collection

**Figure 2:**
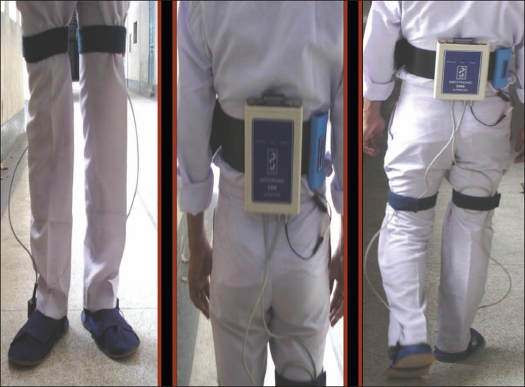
Method of data recording

Each subject was made to wrap an ultraflex unit around the waist and a pair of CDG shoes of approximate size was put on the feet. The subjects were then given two minutes of familiarization time. After the familiarization time the subjects were made to walk at a natural speed, straight, in a ten-meter corridor. Data was than taken for 20 seconds. The recorded data were than transferred to a processor by link cables and were analyzed from the fifth to the 15^th^ seconds of gait as it was supposed to represent natural gait pattern [Figure 2].

Gait parameters and vertical ground reaction forces assessment is done by measuring the following data:

#### Gait cycle properties:

Gait cycle duration, Frequency, Symmetry.

#### Step time Parameter:

Single support time, Double support time, Stance time, Step time and Single swing time.

#### Vertical ground reaction forces variables:

Include first and second peak forces (f1, f2), time to first and second peak force (t1, t2), Loading rate (calculated as magnitude of first peak force divided by the time at which it occurred) and Push-off rate (calculated as the magnitude of second peak force divided by the time from the second peak force until the end of the stance).

#### Data reduction and analysis:

All data was reduced to mean pressure in each sensor by the software in CDG. Only step time parameter and vertical ground reaction forces measures obtained by force graphics and histogram were meticulously noted. Now mean of each group data was calculated and comparison done. For statistical significance ‘t’ value and ‘*P*’ value was calculated. Changes were considered significant when ‘*P*’ value was <. 05.

## RESULTS

All data was noted and comparison was done between affected limb of total arthoplasty group and unaffected limb and control group on the basis of gait cycle properties, step time parameters and vertical ground reaction forces variables obtained by histogram and force graphics.

There were a number of significant differences for gait cycle properties and vertical ground reaction forces between the arthroplasty group and the control group.

### Gait cycle properties:

Frequency was reduced in total arthroplasty group (99.4) as compared to the control group (110.9) while Gait cycle duration increased in total arthoplasty group (1.3145 sec) as compared with control.

### Step time parameters:

Single support time (15.82%), stance time (22.87%) and step time (15.8%) were reduced while single swing time (59.76%) and double support time (64.27%) were increased on affected limb when compared with the control group while all these parameters had increased on the unaffected site except single swing time which had decreased (3.35%) [Table a and b].

**Table a:** Comparison of step time parameters of affected limb with control group

Step time parameter	Control		Affected side		‘t’ value	‘*P*’ value
				
	Mean (Sec)	SD	Mean (Sec)	SD		
Single support time	0.3475	0.025	0.325	0.061	3.230	0.003
Double support time	0.131	0.00	0.2996	0.067	11.169	0.000
Stance time	1.0249	0.003	0.9365	0.069	15.786	0.000
Step time	0.7162	0.003	0.6768.	0.071	8.656	0.000
Single swing time	0.375	0.014	0.94195	0.073	1.450	0.155

**Table b:** Comparison of step time parameters of affected limb with unaffected limb

Step time parameter	Unaffected side		Affected side		‘t’ value	‘*P*’ value
				
	Mean (Sec)	SD	Mean (Sec)	SD		
Single support time	0.3631	0.059	0.325	0.061	2.01	0.003
Double support time	0.2993	0.159	0,2996	0.067	9.269	0.000
Stance time	1.01	0.090	0.9365	0.069	16.74	0.000
Step time	0.6388	0.082	0.6768	0.071	11.345	0.000
Single swing time	0.3913	0.064	0.94195	0.073	3.983	0.155

### Ground reaction forces over each sensor:

Forces reduced on Toe (19.45%), MMF (Midsole medial front, 26.02%), MLF (Midsole lateral front, 19.13%), MMR (Midsole medial rear, 16.99%) and MLR (Midsole lateral rear, 21.6%) on the affected side while increased over all sensors except at heel (38.11%) on the unaffected side [Table c].

**Table c:** Comparison of vertical ground reaction forces over each sensor of affected limb with unaffected limb and control group

Sensors	Control group	Unaffected limb (Mean)	Affected limb (Mean)
Toe	55	44.65	44.3
Midsole medial front	44	31.5	32.75
Midsole lateral front	40.5	35.25	32.75
Midsole medial middle	42	11.65	68.7
Midsole lateral middle	15	18.55	28.5
Midsole medial rear	51.5	36	42.75
Midsole lateral rear	62.5	46.85	49
Heel	22	35.55	39.25

### Ground reaction forces variables measures:

The magnitude of the first and second peak forces was significantly reduced on the affected limb of the arthroplasty group when compared against the data of either their unaffected leg or control group. There were no differences in the peak forces values between the unaffected side of the hip arthroplasty subject and control group. The first peak force occurs at a significantly later time in the stance phase on the affected limb although there were no timing differences between the unaffected and control group. The second peak force occurs at a similar time on both legs in the total arthroplasty group and control group. Loading rate was significantly greater on the unaffected leg of the hip replacement subjects when compared to their affected leg and that of the control group. The push-off rate was greater on the affected leg of the total arthroplasty group as compared to the control group [Table d].

**Table d:** Ground reaction forces variables measures

Vertical ground reaction forces variables	Control group (Mean)	Unaffected limb (Mean)	Affected limb (Mean)
First peak force (N)	1.05	1.06	1.01
Time to first peak force (Sec)	29.0	28.0	33.0
Second peak force (N)	1.02	1.02	1.00
Time to second peak force (Sec)	68.0	69.0	67.0
Loading rate (N / Sec)	5.22	6.21	5.00
Push-off rate (N / Sec)	6.12	6.44	6.69

## DISCUSSION

Previous studies on total hip arthroplasty (THA) have focused on surgical technique and postoperative management or radiographic assessment and not on functional outcome.

This study demonstrates that though there was considerable improvement in all gait parameters, the patients did not improve to a level that would be considered normal for their age group. Gait analysis was done only after six months because it was proved by previous studies that the greatest improvement of gait symmetry and of both temporal and spatial gait parameters (such as stride length and double support time) occurred within the first six months after unilateral THA.

Frequency measured denotes that the walking ability is reduced in the postreplacement group. Gait cycle duration denotes the time elapsed in single gait cycle. Increment in gait cycle duration signifies that the patient is doing more postural adjustment while walking. This also results in reduced frequency. Walking ability after total hip arthroplasty, suggested that although hip movements are pain-free in postreplacement cases they are not normalized according to their age group. This supports our study. Symmetry of left to right foot parameters is not maintained because both hips do not equally share weight-bearing, which is clearly obtained by assessing the step time parameters.

Step time parameters show both weight-bearing duration of a particular limb as well as duration when the limb is off the ground. Single support time, step time and statue time are reduced on the affected limb in both groups while single swing time and double support time increased. This shows that patients avoid weight-bearing on the affected limb and most of the time keep the limb off the ground. Increase in double support time shows that when the patient bears weight on the affected side the contralateral limb also supports it. These changes are significant (*P* value < 0.05).

Assessment of gait parameters clearly shows that the walking ability is reduced in postreplacement patients and they also avoid weight-bearing on the affected limb.

Unequal limb loading was measurable from the ground reaction force curves. The asymmetries can be quantified when the affected and the unaffected legs of the hip replacement subjects are compared against each other and against healthy control subjects.

Both the first and the second vertical force peaks were less on the affected leg when compared with their unaffected leg as well as with control group. This shows that patients put weight on the affected leg in a protected manner.

The time to first peak force occurred significantly later on the affected leg of hip replacement patients; it shows that patients do not put weight on the affected leg as quickly as they do on the unaffected leg.

Loading rate was less and push-off rate was more on the affected limb, which shows that patient, switch over to the unaffected leg as quickly as possible. Although the patients in the arthroplasty group were pain-free, they were favoring their affected limb by not putting as much force on it and not as quickly, during the weight acceptance phase of walking. Adriacchi^8^ hypothesized that individuals with a joint pathology adopt a gait reprogramming their movement patterns.

The current study ascertains that in hip replacement subjects hip strength^9^ and hip range of motion^1^,^9^,^10^ improve after surgery but do not reach normal level and subjects walk with a residual antalgic gait well after surgery.

Although the subjects in the hip arthroplasty group were pain-free, they were favoring their affected limb by not putting as much force on it and not as quickly, during the weight acceptance phase of walking; this results in additional stress on the unaffected leg eventually leading to development of osteoarthritis in that leg.^11^–^14^ Joint degeneration on the healthy contralateral limb seen by Arsever and Bole^11^ and Suter and coworkers^14^ was attributed to increased limb loading. Dekel *et al.*^12^ and Radin^13^ also noted severe articular cartilage degeneration of the knee in the presence of limb overloading.

Prosthetic joints' designs are based on studies of normal gait pattern. If the individual walks with an abnormal gait, this unexpected pattern of wear and tear may lead to mechanical failure of implant. So it is advisable to train these individuals to walk with equal force distribution on both legs. No preoperative data was collected on these subjects. It is therefore unknown if this pattern of walking may be attributed to a residual antalgic gait adopted when these subjects had severe, painful arthritis in the joint.
